# Two-Dimensional Sampling-Recovery Algorithm of a Realization of Gaussian Processes on the Input and Output of Linear Systems

**DOI:** 10.3390/e22101079

**Published:** 2020-09-25

**Authors:** Vladimir Kazakov, Mauro A. Enciso, Francisco Mendoza

**Affiliations:** Departamento Telecomunicaciones, Sección de Posgrado e Investigación, Instituto Politécnico Nacional, Unidad Zacatenco, National Polytechnic Institute of Mexico, Ave. IPN s/n, Building Z, Access 4, 3th Floor, SEPI Telecommunications, Mexico City 07738, Mexico; mauro.enciso@gmail.com (M.A.E.); fcm2709@gmail.com (F.M.)

**Keywords:** sampling recovery algorithm of a realization of multidimensional gaussian process, conditional mean rule, basic function, error recovery function, covariance function, cross-covariance function

## Abstract

Based on the application of the conditional mean rule, a sampling-recovery algorithm is studied for a Gaussian two-dimensional process. The components of such a process are the input and output processes of an arbitrary linear system, which are characterized by their statistical relationships. Realizations are sampled in both processes, and the number and location of samples in the general case are arbitrary for each component. As a result, general expressions are found that determine the optimal structure of the recovery devices, as well as evaluate the quality of recovery of each component of the two-dimensional process. The main feature of the obtained algorithm is that the realizations of both components or one of them is recovered based on two sets of samples related to the input and output processes. This means that the recovery involves not only its own samples of the restored realization, but also the samples of the realization of another component, statistically related to the first one. This type of general algorithm is characterized by a significantly improved recovery quality, as evidenced by the results of six non-trivial examples with different versions of the algorithms. The research method used and the proposed general algorithm for the reconstruction of multidimensional Gaussian processes have not been discussed in the literature.

## 1. Introduction

The list of publications devoted to the study of sampling-recovery algorithms (SRA) for realization of random processes is huge and difficult to read. The problem, formulated in the title of the article, covers issues related to multidimensional SRA. Let us note two of the standard and most important of them: (1) In accordance with the selected criterion, it is necessary to determine the optimal structure of the device for restoring realizations of the selected random process for a given set of samples and (2) to assess the quality of restoration realizations. These two problems must be studied for many types of stochastic processes and for different types of sampling realizations. In the general case, the set of samples of realizations can be random and described by a stream of random points. Deterministic sampling can be periodic or non-periodic. When random jitter or gaps are present in the samples, the determinism of the samples disappears. In addition, the number of samples involved in recovery in all these cases can be arbitrary.

For each of the options mentioned, specific bibliographic lists of published works can be found. Here we will indicate only a few typical publications [1,2,3,4,5,6,7,8,9], in which SRAs of multidimensional stochastic processes are discussed (the list of works does not claim to be complete). A similar situation is due to the fact that in this article: (1) The study is carried out using the conditional mean method (CMM), which has not been used by other authors when solving such problems; (2) the problem of restoring the realizations for individual components of a multidimensional Gaussian process based on given samples of all components, has not been investigated. The conducted bibliographic search for the problem “Theory of sampling” did not reveal sources with the indicated characteristics, with the exception the author’s publications.

The application of the CMM (see, for example, [10,11,12]) to the study of SRA of realizations of random processes has a number of advantages (see [13,14,15,16,17] and references therein) in comparison with the well-known Balakrishnan theorem (TB) [18] and many of its generalizations. Indeed, SRA based on CMM are distinguished by such positive qualities as: (1) Restoration of a sampled realization of a random process according to the CMM automatically provides a minimum of the root-mean-square error of restoration; (2) the restoring function, like the restoring error function in the general case, takes into account the main statistical characteristics of a random process: Probability density, covariance, and cumulant functions; spectrum (a process with a limited spectrum is a special case); (3) the considered algorithms are optimal for any number and location of samples (the variant of periodic samples is a special case); (4) general analytical expressions for the considered SRA cover stationary and non-stationary variants of stochastic processes; (5) sampled stochastic processes can be Gaussian and non-Gaussian, continuous and discontinuous, etc. Moreover, the CMM has been productively applied to study the SRA of random fields, both Gaussian [19,20] and fields with jumps [17,21]. Of course, the version of a multidimensional Gaussian process turns out to be more convenient for analysis since there are simple analytical relations for it.

Note, the application of CMM to the study of SRA-realizations of multidimensional random processes has not been sufficiently discussed in the literature. The work is intended to partially fill this gap. The aim of the article is to study SRA for message models that are described by two-dimensional Gaussian random processes. However, the dimension of the problem is not limited by the presence of two random processes at the input and output of the linear system, since in addition to them, the problem includes two sets of samples fixed in the realizations of these processes. These fixed sets are made up of an arbitrary number of samples that are randomly located on the time axis. The number of samples involved in reconstruction significantly increases the dimension of the problem. CMM expressions for a multidimensional random Gaussian variable [22,23] are generalized in relation to the problem formulated in the title of the article. The use of CMM allows us to overcome the difficulties that arise and obtain general expressions for describing the optimal structures and assessing the quality of the restoration.

In practice, the option under discussion arises, for example, in telemetry systems, when messages with statistical dependence are transmitted over separate channels. The most suitable and convenient model for this kind of messages is a set of Gaussian random processes at the input x(t) and output y(t) of an arbitrary linear system described by an impulse response h(t). By changing the type of the function h(t), you can change the type of statistical relationship between the two processes. In this case, the message is a two-dimensional Gaussian process ${\left[x\left(t\right),y\left(t\right)\right]}^{T}$. The realizations of both components are sampled and transmitted to the receiving side. Sets of samples $X\left(T^{\left(x\right)}\right)$ and $Y\left(T^{\left(y\right)}\right)$ realizations of components x(t) and y(t) are arbitrary both in quantity and location on the time axis.

The matrix description of the recovery procedure allows one to obtain general optimal recovery algorithms for both input and output realizations using both sets of samples $X\left(T^{\left(x\right)}\right)$ and $Y\left(T^{\left(y\right)}\right)$. In addition, you can evaluate the quality of restoration of the realizations of both components. In this case, the recovery of each of the realizations by the proposed method turns out to be higher than with the usual recovery algorithm only from its own samples. The study of this general case allows us to consider two particular options, each of which consists in the fact that on the basis of both sets of samples $X\left(T^{\left(x\right)}\right)$ and $Y\left(T^{\left(y\right)}\right)$ it is necessary to restore only one of the transmitted messages. For example, the algorithm for restoring the realization of the output process y(t) must use not only a set of its own samples $Y\left(T^{\left(y\right)}\right)$, but also a set of samples to implement an auxiliary input process $X\left(T^{\left(x\right)}\right)$. The examples below also consider the opposite case, when the realization of the input process is restored, and the samples of the realization of the output process play an auxiliary role. The positive effect of this operation is due to the fact both processes are statistically related, and therefore the role of the cross-covariance function between output and input is significant.

The aim of the study is to provide an analytical description of the proposed algorithm for recovering realizations of a two-dimensional Gaussian process and to assess the quality of its functioning, taking into account the sets of samples of realizations of both processes. It should be emphasized that instead of analyzing reconstruction functions that depend on a set of sample values, we will study basic functions, which are the impulse responses of the shaping filters for each sample. Reconstruction functions are created by multiplying each sample by its own basic function and then adding them. It is clear the basic functions are independent of the sampled values.

The scientific novelty of the article is as follows: (1) The sampling—restoration algorithms (SRA) of realizations of the components of a two-dimensional Gaussian process are studied, taking into account the fact that the restoration is carried out not only on the basis of a set of their own samples of the realization of the selected component, but also taking into account the set of samples of the realization of another component, statistically related to the first. Owing to the use of CMM, general variants of SRAs with an arbitrary number and location of samples in both realizations are investigated. (2) As a result, a general scheme of the restorer of both realizations was obtained, which provides minimal restoration errors. In addition, general relations are found to estimate the minimum recovery errors for each of the sampled realizations. (3) For several typical models of linear systems with different input processes, the cross-covariance functions between input and output are determined. These functions play a major role in studying the influence of the set of samples of the realization of the auxiliary component on the structure and quality of restoration of the realization of the selected component. (4) Variants of SRA have been studied when the restoration of the realization of the selected component occurs at one or several sampling intervals for various cross-covariance functions of the processes. Several examples investigate non-trivial cases when the sampling intervals of the realizations of both components are different. (5) In all the options considered, the optimal forms of the basic functions are determined, and the functions of recovery errors are calculated. The latter show the advantages of the proposed method in improving the quality of restoration in comparison with the classical method when restoration is carried out only according to own samples of realization.

The article consists of the following sections. Section 2 presents general formulas for the vector of conditional mathematical expectations and the matrix of conditional covariances in relation to the here considered. Section 3 discusses the models of the Gaussian processes used. Section 4 is devoted to the description of the optimal structure of the reductant of realizations of both components of a two-dimensional Gaussian process based on a set of samples of the input and output processes of an arbitrary linear system. Section 5 discusses examples describing SRP in a single recovery interval. Section 6 is devoted to examining SRP at multiple recovery intervals. There is one Appendix A.

## 2. General Formulas for the Statistical Characteristics of the Two-Dimensional Conditional Gaussian Process

In the mathematical literature, there is a result that is closely related to the problem formulated in here. Namely, in [22] (see also [23]), matrix expressions were obtained for the conditional mean vector and for the conditional covariance matrix of one vector for a fixed other vector. These relations have been derived for multidimensional Gaussian random variables. These formulas are given in the Appendix A and are designated by the letter “A”. They cannot be used directly to solve the problem posed in the article. We generalize them to the case when two components are Gaussian processes with continuous time, and the other components (sets of samples) are random Gaussian variables with discrete time.

For our purpose, we use different designations than those used in the Appendix A. Consider a column vector $Z\left(t,T^{\left(x\right)},T^{\left(y\right)}\right)$ that is analogous to the vector **z** (see Formula (A1)):

(1) $$Z\left(t,T^{\left(x\right)},T^{\left(y\right)}\right)={\left[Z_{1}\left(t\right),Z_{2}\left(T^{\left(x\right)},T^{\left(y\right)}\right)\right]}^{T}$$

(2) $$Z_{1}\left(t\right)={\left[x\left(t\right),y\left(t\right)\right]}^{T}$$

(3) $$Z_{2}\left(T^{\left(x\right)},T^{\left(y\right)}\right)={\left[X\left(T^{\left(x\right)}\right),Y\left(T^{\left(y\right)}\right)\right]}^{T},$$

where

(4) $$X\left(T^{\left(x\right)}\right)={\left[x_{1}\left(T_{1}^{\left(x\right)}\right),x_{2}\left(T_{2}^{\left(x\right)}\right),\ldots ,x_{N^{\left(x\right)}}\left(T_{N^{\left(x\right)}}^{\left(x\right)}\right)\right]}^{T},$$

(5) $$Y\left(T^{\left(y\right)}\right)={\left[y_{1}\left(T_{1}^{\left(y\right)}\right),y_{2}\left(T_{2}^{\left(y\right)}\right),\ldots ,y_{N^{\left(y\right)}}\left(T_{N^{\left(y\right)}}^{\left(y\right)}\right)\right]}^{T}$$

where $N^{\left(x\right)},N^{\left(y\right)}$ are the numbers of samples in both sets.

The vector $Z\left(t,T^{\left(x\right)},T^{\left(y\right)}\right)$ is described by the mathematical expectation vector (see analogue the Formula (A2)):

(6) $$m\left(t,T^{\left(x\right)},T^{\left(y\right)}\right)={\left[\langle Z_{1}\left(t\right)\rangle ,\langle Z_{2}\left(T^{\left(x\right)},T^{\left(y\right)}\right)\rangle \right]}^{T}$$

(7) $$\langle Z_{1}\left(t\right)\rangle ={\left[m_{x}\left(t\right),m_{y}\left(t\right)\right]}^{T}$$

(8) $$\langle Z_{2}\left(T^{\left(x\right)},T^{\left(y\right)}\right)\rangle ={\left[\langle X\left(T^{\left(x\right)}\right)\rangle ,\langle Y\left(T^{\left(y\right)}\right)\rangle \right]}^{T}$$

and covariance matrix

(9) $$K\left(t,t^{\prime },T^{\left(x\right)},T^{\left(y\right)}\right)=\left[\begin{array}{cc} K_{11}\left(t,t^{\prime }\right) & K_{12}\left(t,T^{\left(x\right)},T^{\left(y\right)}\right)\\ K_{21}\left(T^{\left(y\right)},T^{\left(x\right)},t^{\prime }\right) & K_{22}\left(T^{\left(x\right)},T^{\left(y\right)}\right) \end{array}\right]$$

where $K_{11}\left(t,t^{\prime }\right),K_{22}\left(T^{\left(x\right)},T^{\left(y\right)}\right)$ are the covariance matrices of vectors $Z_{1}\left(t\right)$ and $Z_{2}\left(T^{\left(x\right)},T^{\left(y\right)}\right)$, respectively; $K_{12}\left(t,T^{\left(x\right)},T^{\left(y\right)}\right),K_{21}\left(T^{\left(y\right)},T^{\left(x\right)},t^{\prime }\right)$—matrices of cross covariance between vectors $Z_{1}\left(t\right)$ and $Z_{2}\left(T^{\left(x\right)},T^{\left(y\right)}\right)$. Expression (9) is an analog of the matrix (A3) written in the new notation. We fix the vector $Z_{2}\left(T^{\left(x\right)},T^{\left(y\right)}\right)$, and the vector ${\tilde{Z}}_{1}\left(t\right)$ remains random with its components conditional with respect to the vector $Z_{2}\left(T^{\left(x\right)},T^{\left(y\right)}\right)$. Then, the vector ${\tilde{Z}}_{1}\left(t\right)$ is described by a Gaussian two-dimensional conditional probability density, which is characterized by a column vector of conditional mathematical expectations and a matrix of conditional covariances. The vector of conditional mathematical expectations instead of (A4) is written in the form:

(10) $$\begin{array}{c} \langle {\tilde{Z}}_{1}\left(t\right)\rangle =\langle Z_{1}\left(t|X\left(T^{\left(x\right)}\right),Y\left(T^{\left(y\right)}\right)\right)\rangle =\langle Z_{1}\left(t\right)\rangle +\\ +K_{12}\left(t,T^{\left(x\right)},T^{\left(y\right)}\right)K_{22}^{-1}\left(T^{\left(x\right)},T^{\left(y\right)}\right)\left(Z_{2}(T^{\left(x\right)},T^{\left(y\right)})-\langle Z_{2}(T^{\left(x\right)},T^{\left(y\right)})\rangle \right) \end{array}$$

As in the one-dimensional case [13,14,15,16], based on (10), we introduce the definition of a multidimensional basic function

(11) $$\begin{array}{l} b\left(t,T^{\left(x\right)},{Τ}^{\left(y\right)}\right)={\left[b^{\left(x\right)}\left(t,T^{\left(x\right)},{Τ}^{\left(y\right)}\right),b^{\left(y\right)}\left(t,T^{\left(x\right)},{Τ}^{\left(y\right)}\right)\right]}^{T}=\\ =K_{12}\left(t,T^{\left(x\right)},T^{\left(y\right)}\right)K_{22}^{-1}\left(T^{\left(x\right)},T^{\left(y\right)}\right) \end{array}$$

Relation (10) determines the optimal recovery structure for the sampled realizations of the two-dimensional process (see the Section 4). Recovery should be carried out sequentially at sampling intervals.

The matrix of conditional covariance $\tilde{K}\left(t,t^{\prime }|T^{\left(x\right)},T^{\left(y\right)}\right)$ of the vector function $\tilde{Z}\left(t|T^{\left(x\right)},T^{\left(y\right)}\right)$, when the vector $Z_{2}\left(T^{\left(x\right)},T^{\left(y\right)}\right)$ is fixed, based on (A5), (9) takes the form:

(12) $$\tilde{K}\left(t,t^{\prime }|T^{\left(x\right)},T^{\left(y\right)}\right)=K_{11}\left(t,t^{\prime }\right)-K_{12}\left(t,T^{\left(x\right)},T^{\left(y\right)}\right)K_{22}^{-1}\left(T^{\left(x\right)},T^{\left(y\right)}\right)K_{21}\left(T^{\left(x\right)},T^{\left(y\right)},t^{\prime }\right)$$

Equating in (12) times $t^{\prime }=t$, it is possible to obtain relations that determine the functions of conditional variance, which characterize the quality of restoration of realizations of each component.

Let us describe the general form of the submatrices included in expression (9). The two-dimensional Gaussian process ${\left[\begin{array}{cc} x\left(t\right), & y\left(t\right) \end{array}\right]}^{T}$ is described by the mathematical expectation vector (7) and the covariance matrix

(13) $$K_{11}\left(t,t^{\prime }\right)=\left[\begin{array}{cc} K_{x}\left(t,t^{\prime }\right) & K_{xy}\left(t,t^{\prime }\right)\\ K_{yx}\left(t,t^{\prime }\right) & K_{y}\left(t,t^{\prime }\right) \end{array}\right]$$

In (13), functions $K_{x}\left(t,t^{\prime }\right),K_{y}\left(t,t^{\prime }\right)$ are covariance functions of processes x(t) and y(t), accordingly. The degree of statistical dependence between the processes is determined by the functions of cross covariance $K_{xy}\left(t,t^{\prime }\right),K_{yx}\left(t,t^{\prime }\right)$. The remaining three sub-matrices are written this way:

(14) $$K_{12}\left(t,T^{\left(x\right)},T^{\left(y\right)}\right)=\left[\begin{array}{cc} \langle \dot{x}\left(t\right)\dot{X}\left(T^{\left(x\right)}\right)\rangle & \langle \dot{x}\left(t\right)\dot{Y}\left(T^{\left(y\right)}\right)\rangle \\ \langle \dot{y}\left(t\right)\dot{X}\left(T^{\left(x\right)}\right)\rangle & \langle \dot{y}\left(t\right)\dot{Y}\left(T^{\left(y\right)}\right)\rangle \end{array}\right]$$

Here and below, the dots above the letters indicate centered random variables.

(15) $$K_{21}\left(T^{\left(x\right)},T^{\left(y\right)},t^{\prime }\right)=\left[\begin{array}{cc} \langle \dot{X}\left(T^{\left(x\right)}\right)\dot{x}\left(t^{\prime }\right)\rangle & \langle \dot{X}\left(T^{\left(x\right)}\right)\dot{y}\left(t^{\prime }\right)\rangle \\ \langle \dot{Y}\left(T^{\left(y\right)}\right)\dot{x}\left(t^{\prime }\right)\rangle & \langle \dot{Y}\left(T^{\left(y\right)}\right)\dot{y}\left(t^{\prime }\right)\rangle \end{array}\right]$$

(16) $$K_{22}\left(T^{\left(x\right)},T^{\left(y\right)}\right)=\left[\begin{array}{cc} \langle \dot{X}\left(T^{\left(x\right)}\right)\dot{X}\left(T^{\left(x\right)}\right)\rangle & \langle \dot{X}\left(T^{\left(x\right)}\right)\dot{Y}\left(T^{\left(y\right)}\right)\rangle \\ \langle \dot{Y}\left(T^{\left(y\right)}\right)\dot{X}\left(T^{\left(x\right)}\right)\rangle & \langle \dot{Y}\left(T^{\left(y\right)}\right)\dot{Y}\left(T^{\left(y\right)}\right)\rangle \end{array}\right]$$

Using Formulas (13)—(16), we can specify the relations (10) and (12), which should be calculated sequentially in the intervals for interpolation $T_{i-1}<t\leq T_{i},i=2,3,\ldots ,N$ and for extrapolation at $t\geq T_{N}$. There is a retropolation option, when $t\leq T_{1}$. Here, the superscripts (x) and (y) are omitted.

## 3. Models of Used Gaussian Processes

Below, the use of the above general algorithm is illustrated with a series of examples in which two statistically related Gaussian processes appear. Covariance and cross-covariance functions of processes vary within wide limits. As indicated in Section 1, these processes are most simply described using a linear system with a given impulse response h(t). When the covariance function $K_{x}\left(\tau \right)$ of the input process x(t) and characteristics h(t) change, the output process y(t) is described by various covariance functions. In this case, of course, the cross-covariance function between the input and output is also changed. There are general formulas [23], which can be used to determine the desired covariance functions for given $K_{x}\left(\tau \right)$ and h(t). Let us write them out in relation to the stationary case, setting $m_{x}\left(t\right)=m_{y}\left(t\right)=0$:

(17) $$K_{xy}\left(\tau \right)=\int_{0}^{\infty }h\left(u\right)K_{x}\left(\tau -u\right)du,\tau =t-t^{\prime }$$

There are two cross-covariance functions $K_{xy}\left(\tau \right),K_{yx}\left(\tau \right)$, that have the property $K_{xy}\left(\tau \right)=K_{yx}\left(-\tau \right)$:

(18) $$\begin{array}{l} K_{y}\left(\tau \right)=\int_{0}^{\infty }\int_{9}^{\infty }h\left(u_{1}\right)h\left(u_{2}\right)K_{x}\left(\tau -u_{2}+u_{1}\right)du_{1}du_{2}=\\ =\int_{0}^{\infty }h\left(u\right)du\int_{-\infty }^{\tau +u}h\left(\tau +u-v\right)K_{x}\left(v\right)dv \end{array}$$

For our purposes, when choosing linear systems, it is advisable to choose the simplest in structure and description. In this case, it can easily be demonstrated how the auto- and cross-covariance functions of the input and output processes of linear systems affect the main characteristics of the SRP: The structure of recovery devices (or basic functions) and the functions of recovery errors. As linear systems, it is appropriate to choose low-pass filters, consisting of series-connected integrating RC circuits, at the input of which there is white Gaussian noise. Such systems can have one or more connected integrating RC circuits separated by buffer cascades [23]. At the outputs of such systems, Gaussian processes with various statistical characteristics are formed. Below, this method will be used to describe both input and output processes.

The simplest linear system is a single integrating RC circuit, at the input of which there is white noise. At the output of such a system, a Markov Gaussian process with an exponential covariance function is formed. At the outputs of two, three, and further circuits, the output processes will not be Markov.

Formulas (17) and (18) will be used below when considering examples.

## 4. General Optimal Structure of Restoration of Realizations of the Two-Dimensional Gaussian Process

**Figure 1:**
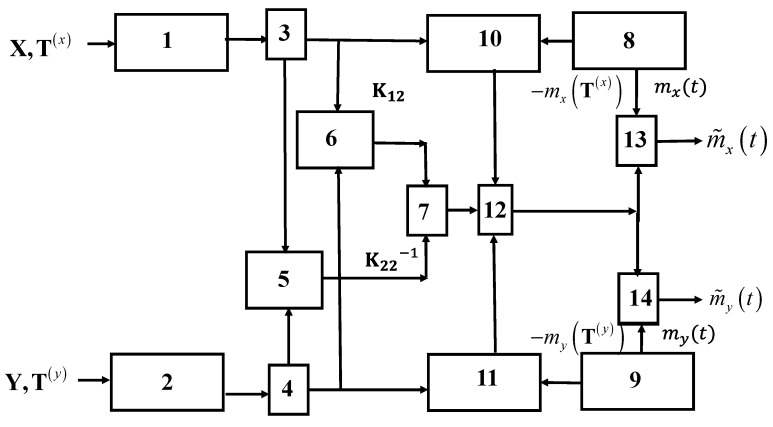
General scheme of recovery of two-dimensional process realizations.

Optimal recovery is understood to mean an algorithm that uses both sets of samples $X\left(T^{\left(x\right)}\right),Y\left(T^{\left(y\right)}\right)$ in the recovery of each of the components of a two-dimensional process $Z_{1}\left(t\right)={\left[x\left(t\right),y\left(t\right)\right]}^{T}$. The structure of the optimal recovery device is determined by Formula (10) and is given in Figure 1.

Both inputs of the device receive sets of samples $X\left(T^{\left(x\right)}\right),Y\left(T^{\left(y\right)}\right)$, which are stored in memory registers 1 and 2. The sets of samples are shifted in blocks 3 and 4 to obtain the best restoration quality (see, for example, Example 3). Then, information about the location of the samples along with the characteristics of the linear system is used to calculate the matrix elements $K_{12}\left(t_{1},T^{\left(x\right)},T^{\left(y\right)}\right),K_{22}^{-1}\left(T^{\left(y\right)},T^{\left(x\right)}\right)$ in blocks 5 and 6. In block 7, these matrices are multiplied. A priori information about the mathematical expectation functions (7) and (8) is stored in blocks 8, 9, and is used when subtracting average values $m_{x}\left(T^{\left(x\right)}\right),m_{y}\left(T^{\left(y\right)}\right)$ at the reference points in blocks 10, 11, and also when summing the functions $m_{x}\left(t\right),m_{y}\left(t\right)$ in blocks 13, 14 In block 12, matrix multiplication is performed from the output of block 7 and elements of a centered a column vector of input samples. Recovered realizations ${\tilde{m}}_{x}\left(t\right),{\tilde{m}}_{y}\left(t\right)$ are formed at outputs of blocks 13, 14.

We draw attention to the fact that the matrix of basic functions $b\left(t,T^{\left(x\right)},{Τ}^{\left(y\right)}\right)$ in the diagram in Figure 1 is not indicated. However, in accordance with (11), it is formed at the output of block 7. The elements of the matrix $b\left(t,T^{\left(x\right)},{Τ}^{\left(y\right)}\right)$ represent a set of an orthonormal system of functions. It means that

(19) $$b_{k}^{}\left(t=T_{j}^{}\right)=\{\begin{array}{c} 1,k=j\\ 0,k\neq j \end{array};k,j=1,2,\ldots ,N^{\left(x\right)}+N^{\left(y\right)}$$

superscripts are omitted here.

The number of basic functions is the same as the total number of samples. To clarify the physical meaning of the elements of the matrix $b\left(t,T^{\left(x\right)},{Τ}^{\left(y\right)}\right)$, consider a special case when $N^{\left(x\right)}=N^{\left(y\right)}=2$. This option is explored in the Examples 1 and 2 in the next section. Let us concretize the matrices included in relation (11):

(20) $$K_{12}\left(t,T^{\left(x\right)},T^{\left(y\right)}\right)=\left[\begin{array}{cccc} K_{xx}\left(t,T_{1}^{\left(x\right)}\right) & K_{xx}\left(t,T_{2}^{\left(x\right)}\right) & K_{xy}\left(t,T_{1}^{\left(y\right)}\right) & K_{xy}\left(t,T_{2}^{\left(y\right)}\right)\\ K_{yx}\left(t,T_{1}^{\left(x\right)}\right) & K_{yx}\left(t,T_{2}^{\left(x\right)}\right) & K_{yy}\left(t,T_{1}^{\left(y\right)}\right) & K_{yy}\left(t,T_{2}^{\left(y\right)}\right) \end{array}\right]$$

(21) $$K_{22}^{-1}\left(T^{\left(x\right)},T^{\left(y\right)}\right)=\left[\begin{array}{cccc} a_{11} & a_{12} & a_{13} & a_{14}\\ a_{21} & a_{22} & a_{23} & a_{24}\\ a_{31} & a_{32} & a_{33} & a_{34}\\ a_{41} & a_{42} & a_{43} & a_{44} \end{array}\right]$$

As a result of multiplying (20) and (21), we obtain the matrix of basic functions

(22) $$b\left(t,T^{\left(x\right)},T^{\left(y\right)}\right)=\left[\begin{array}{cccc} b_{11}\left(t\right) & b_{12}\left(t\right) & b_{13}\left(t\right) & b_{14}\left(t\right)\\ b_{21}\left(t\right) & b_{22}\left(t\right) & b_{23}\left(t\right) & b_{24}\left(t\right) \end{array}\right]$$

whose elements are written in the form (we give only two of them):

(23) $$b_{12}\left(t\right)=K_{xx}\left(t,T_{1}^{\left(x\right)}\right)a_{12}+K_{xx}\left(t,T_{2}^{\left(x\right)}\right)a_{22}+K_{xy}\left(t,T_{1}^{\left(y\right)}\right)a_{32}+K_{xy}\left(t,T_{2}^{\left(y\right)}\right)a_{42}$$

(24) $$b_{23}\left(t\right)=K_{yx}\left(t,T_{1}^{\left(x\right)}\right)a_{13}+K_{yx}\left(t,T_{2}^{\left(x\right)}\right)a_{23}+K_{yy}\left(t,T_{1}^{\left(y\right)}\right)a_{33}+K_{yx}\left(t,T_{2}^{\left(y\right)}\right)a_{43}$$

Let us change the notation:

(25) $$b_{1j}\left(t\right)=b_{j}^{\left(x\right)}\left(t\right),b_{2j}\left(t\right)=b_{j}^{\left(y\right)}\left(t\right),j=\bar{1,4}$$

Using relations (22)–(25), we write expressions for basic functions in the form:

(26) $$b_{j}^{\left(x\right)}\left(t\right)=\sum_{i=1}^{N^{\left(x\right)}}K_{xx}\left(t,T_{i}^{\left(x\right)}\right)a_{ij}+\sum_{p=N^{\left(x\right)}+1}^{N^{\left(x\right)}+N^{\left(y\right)}}K_{yx}\left(t,T_{p-N^{\left(x\right)}}^{\left(y\right)}\right)a_{pj}$$

(27) $$b_{j}^{\left(y\right)}\left(t\right)=\sum_{p=N^{\left(y\right)}+1}^{N^{\left(y\right)}+N^{\left(x\right)}}K_{xy}\left(t,T_{p-N^{\left(y\right)}}^{\left(x\right)}\right)a_{pj}+\sum_{i=1}^{N^{\left(y\right)}}K_{yy}\left(t,T_{i}^{\left(y\right)}\right)a_{ij}$$

Formulas (26) and (27) allow us to give a physical interpretation: (1) As in the one-dimensional case, the basic function for each sample $x\left(T_{j}^{\left(x\right)}\right)$ or $y\left(T_{j}^{\left(y\right)}\right)$ in the general case is determined by the sum of a product of the autocovariance function with arguments $t,T_{k},k=1,2,\ldots ,N$ (superscripts are omitted here) and elements of inverse covariance matrix. The difference is that in the case under consideration, we mean not only autocovariance functions $K_{xx}\left(\cdot \right),K_{yy}\left(\cdot \right)$, but also cross-covariance functions $K_{xy}\left(\cdot \right),K_{yx}\left(\cdot \right)$. (2) It is clear for independent components, the sums with cross-covariances in (26) and (27) disappear. Then, formulas for the basic functions coincide with the expressions for the one-dimensional version, and the diagram in Figure 1 is split into two independent channels.

Each example presented in the article is illustrated not only by the type of basic functions, but also by the corresponding graphs of recovery errors. Moreover, in the latter case, among many curves, a curve corresponding to the reconstruction algorithm is necessarily shown, in which only the own samples of the reconstructed realization are used. Comparison of the quality of restoration is performed for the same process models and selected parameters. Note that the always-proposed algorithm is characterized by an improvement in the quality of functioning.

## 5. Study Cases: Reconstruction of Realizations on One Sampling Interval

Shown in Figure 1, the general recovery scheme includes the option under consideration (one sampling interval) as a special case; therefore, a somewhat simplified scheme will not be discussed. Two of the most important characteristics of the SRA are detailed below: The basic functions for each sample involved in recovery and the error recovery functions. The purpose of considering a set of examples is to find out how the following parameters affect the specified characteristics: (1) The number and location of samples of input and output realizations, (2) input and output covariance functions, (3) their cross-covariance functions, and (4) the type of recovery procedure—on one interval or multiple intervals.

Further research requires specification of data on the number and location of samples. We note one important feature of the discussed algorithm, which will be considered when calculating the recovery errors in all the examples considered below. Formulas (10) and (12) are of a general nature, and their application for a large set of samples is associated with the complication of the device. Theoretically, each sample should participate in the formation of the output processes of the system shown in Figure 1. Actually, the samples of the realization of one component (say x(t)) affect the formation and the error of recovery of the other component y(t) only when the localization of samples of the first component is located near or inside the sampling interval of the recovered realization of the second component. The reason for this effect is that it is realized through the cross-covariance function: When the argument of this function is less than the covariance time $\tau_{c}^{\left(y\right)}$ of the output process $\left(T_{j}^{\left(x\right)}-T_{i}^{\left(y\right)}<\tau_{c}^{\left(y\right)}\right)$, then the value of the function $K_{xy}\left(\tau \right)$ is close to the maximum and the influence of the corresponding sample on the quality of recovery is significant. In addition to the position of the maximum of the function $K_{xy}\left(\tau \right)$, the discrepancy between the samples of the auxiliary and recovered realizations also affect the reduction of the recovery error. Such an effect occurs, for example, with unequal sampling periods $T_{j}^{\left(x\right)}=T_{i}^{\left(y\right)}+\Delta T_{j}$ (see Examples 5 and 6). In this case, the minimum of the recovery error will be in the interval close to the point $\Delta T_{j}+t_{\max}$ (here $t_{\max}$ is location of the point at which the function $K_{xy}\left(\tau \right)$ reaches its maximum within the sampling interval $T_{j}^{\left(y\right)}-T_{j+1}^{\left(y\right)}$). The main characteristics of the SRA are also influenced by the elements of the inverse covariance matrix. However, it is difficult to establish at least some patterns of such influence.

In Section 5.1 Example 1 and Section 5.2 Example 2, the numbers of samples are equal to two and the samples of both realizations are located at the same points. In Section 5.3 Example 3, the auxiliary sample is one, but its location varies within the sampling interval $T_{i}^{\left(y\right)}-T_{i-1}^{\left(y\right)}$.

### 5.1. Example 1. System from One RC Chain with Markov Input Process

A Markov Gaussian process is formed at the output of an integrating RC circuit that is under the influence of white noise. Its normalized covariance function $R\left(\tau \right)=K\left(\tau \right)/\sigma^{2}$ in the stationary mode is determined by the formula

(28) $$R_{x}\left(\tau \right)=\exp\left(-\alpha \left|\tau \right|\right)$$

where α=1/RC is the constant parameter.

We put $m_{x}\left(t\right)=m_{y}\left(t\right)=0$. The linear system is also an integrating RC circuit with an impulse response

(29) h(t)=βexp(−βt)

**Figure 2:**
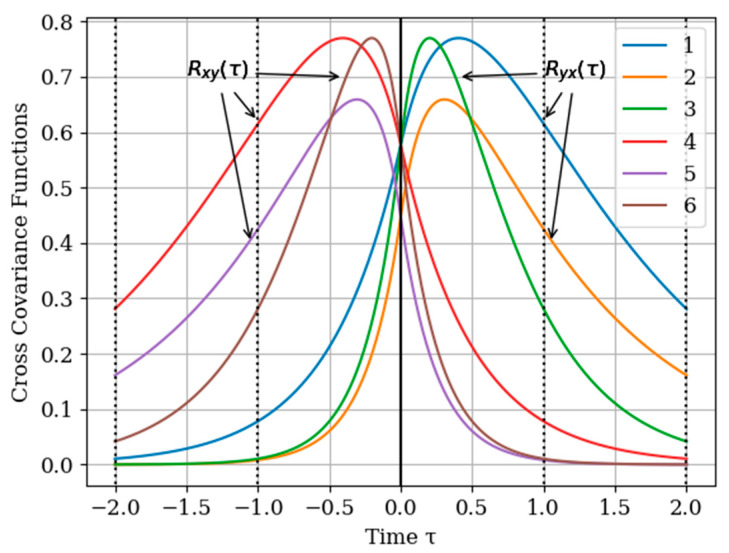
The functions of cross covariance $R_{xy}\left(\tau \right)$ and $R_{yx}\left(\tau \right)$ at various parameter values and α and β.

Using expressions (18), (28) and (29) we determine the normalized covariance function of the output process y(t):

(30) $$R_{y}\left(\tau \right)=\frac{1}{\beta -\alpha }\left[\beta \exp\left(-\alpha \left|\tau \right|\right)-\alpha \exp\left(-\beta \left|\tau \right|\right)\right]$$

as well as normalized cross-covariance functions (17) between the processes x(t) and y(t):

(31) $$R_{xy}\left(\tau \right)=\{\begin{array}{c} \sqrt{\frac{\beta }{\alpha +\beta }}\frac{1}{\beta -\alpha }\left[\exp\left(-\alpha \tau \right)-\frac{2\alpha }{\alpha +\beta }\exp\left(-\beta \tau \right)\right],\tau \geq 0\\ \sqrt{\frac{\beta }{\alpha +\beta }}\frac{1}{\beta +\alpha }\exp\left(-\alpha \tau \right),\tau <0 \end{array}$$

(32) $$R_{yx}\left(\tau \right)=\{\begin{array}{c} \sqrt{\frac{\beta }{\alpha +\beta }}\frac{1}{\beta +\alpha }\exp\left(\alpha \tau \right),\tau \geq 0\\ \sqrt{\frac{\beta }{\alpha +\beta }}\frac{1}{\beta -\alpha }\left[\exp\left(-\alpha \tau \right)-\frac{2\alpha }{\alpha +\beta }\exp\left(-\beta \tau \right)\right],\tau <0 \end{array}$$

where $R_{ij}\left(\tau \right)=K_{ij}\left(\tau \right)/\sigma_{i}\sigma_{j}$. In Figure 2 shows the graphs of the cross covariance function $R_{xy}\left(\tau \right)$ and $R_{yx}\left(\tau \right)$ for various values of the parameters α and β. The curves are calculated for the following parameters: Curve 1—α=2,β=1; curve 2—α=4,β=1; curve 3—α=4,β=2 for $R_{yx}\left(\tau \right)$ and curve 4—α=2,β=1; curve 5—α=4,β=1; curve 6—α=4,β=2 for $R_{xy}\left(\tau \right)$. As can be seen, the cross-covariance functions are odd, and their maxima are shifted of the point τ=0. Especially we note the curves 3 and 4 with their maxima in points τ=0.25 and τ=−0.25 for $R_{xy}\left(\tau \right)$ and $R_{yx}\left(\tau \right)$, respectively. In general, when the value of parameters α,β of cross covariance functions $R_{xy}\left(\tau \right),R_{yx}\left(\tau \right)$ increase, their maxima values decrease. This is explained, because the realizations of the input and output process are more chaotic when the bandwidth is increased, which is described by the value of parameters α,β.

**Figure 3:**
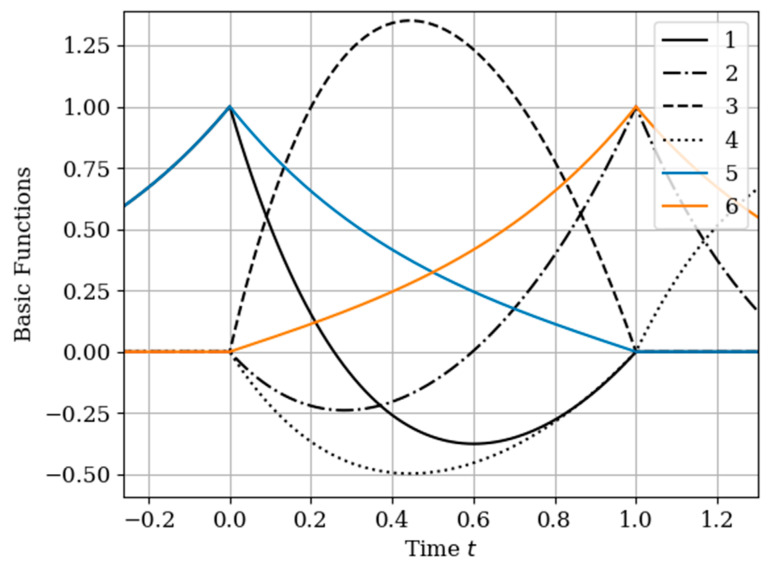
The basic functions of recovery of the realization x(t) in Section 5.1 Example 1.

The results of calculations of basic functions carried out according to formula (11) are shown in Figure 3. The values of the selected parameters are as follows: $N^{\left(x\right)}=N^{\left(y\right)}=2;$ the number of samples involved in the recovery of realizations is the same: The samples are located at the same points: $T_{1}^{\left(x\right)}=T_{1}^{\left(y\right)}=0.0;T_{2}^{\left(x\right)}=T_{2}^{\left(y\right)}=1.0;\alpha =2,\beta =1;\sigma_{x}^{2}=1>\sigma_{y}^{2}$. In Figure 3 shows the basic functions of the multidimensional algorithm $b_{j}^{\left(x\right)}\left(t\right),\left(j=1,\ldots ,N^{\left(x\right)}+N^{\left(y\right)}\right)$ (curves 1–4) and the one-dimensional algorithm $b_{i}\left(t\right),\left(i=1,\ldots ,N^{\left(x\right)}\right)$ (curves 5 and 6). These basic functions correspond to the restoration of realization of process x(t) at the input of the system. The samples of the realization of the output process y(t) are auxiliary samples here. The multivariate algorithm has four basic functions (for own and for auxiliary samples), while the unidimensional algorithm has two basic functions.

Curves 5 and 6 in Figure 3 refer to a one-dimensional algorithm. They are described by the first term in (26) and the covariance function (28). In accordance with (26), the multidimensional algorithm includes four basic functions, including two of them formed on the basis cross covariance functions (32). Moreover, these functions, elements of the inverse matrix, influence the calculation of the basic functions. It is obvious that the form of the multidimensional basic function changes radically in relation to the main functions in a one-dimensional algorithm.

**Figure 4:**
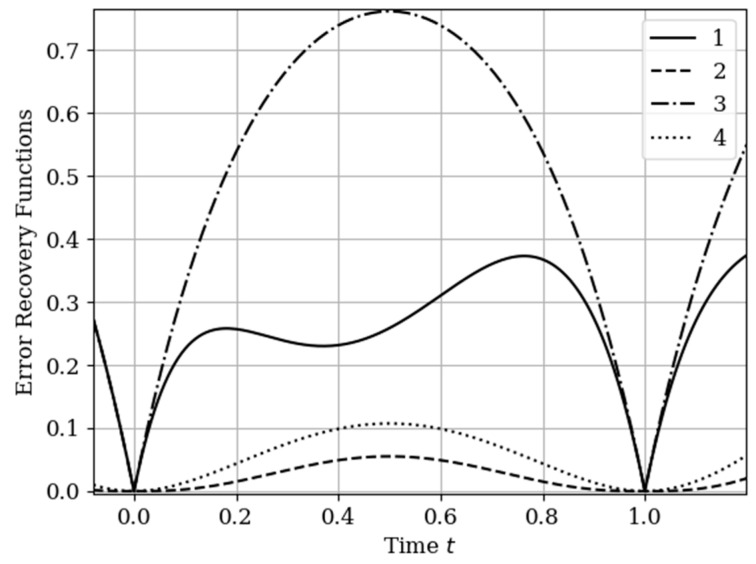
Recovery errors in Section 5.1 Example 1.

The results of calculations of recovery errors carried out according to formula (12) are shown in Figure 4. The values of the selected parameters are the same as in the comments to Figure 3. Curve 1 describes the recovery error of realization of x(t) with multidimensional algorithm. It has a smoothed minimum close to the point τ=0.25, because the function $K_{yx}\left(\tau \right)$ has maximum at this point. The smoothness of the discussed extremum is influenced by the proximity of the control point, where the error is zero by the definition.

Curves 3 and 4 describe the recovery errors ${\tilde{\sigma }}_{x}^{2}\left(t\right),{\tilde{\sigma }}_{y}^{2}\left(t\right)$ for the one-dimensional algorithm, when the recovery is performed only on their own samples. The difference in the values of the curve maxima is explained by the difference in the time structure of the processes: The output process y(t) is smoother than the input process x(t). Curves 1 and 2 are obtained by a multidimensional algorithm, when both sets of samples participate in the restoration of each realization. The even form of curve 3 is explained, because this form is determined by the covariance functions $R_{xy}\left(\tau -T_{1}^{\left(y\right)}\right),R_{xy}\left(\tau -T_{2}^{\left(y\right)}\right)$. According to formula (27), the influential of these functions are weighed by the elements of the inverse matrix. A comparison of pairs of curves 2, 4, and 1, 3 indicates that the restoration using the multidimensional algorithm provides a higher quality of recovery than the similar procedure according to the one-dimensional algorithm.

### 5.2. Example 2. The Input Is Non-Markovian Process Formed by Two Sequential RC Chains. System Is One RC Chain

There is one difference between Example 1 and Example 2: Here, the input process is not Markovian. This circumstance changes all the covariance functions included in the expressions for the analysis of the studied algorithm.

The covariance function of the input process is determined by relation (30) with the change of index.

The linear system under study is described by the function

(33) h(t)=γexp(−γt),                t≥0

and the process at its output is characterized by the covariance function (18)

(34) $$\begin{array}{l} R_{y}\left(\tau \right)=\frac{\beta \left(\gamma -\beta \right)\left(\gamma +\beta \right)\left(\gamma \exp\left(-\alpha \left|\tau \right|\right)-\alpha \exp\left(-\gamma \left|\tau \right|\right)\right)}{\left(\gamma -\alpha \right)\left(\gamma -\beta \right)\left(\beta \left(\gamma +\alpha \right)-\alpha \left(\gamma +\beta \right)\right)}-\\ -\frac{\alpha \left(\gamma -\alpha \right)\left(\gamma +\alpha \right)\left(\gamma \exp\left(-\beta \left|\tau \right|\right)-\beta \exp\left(-\gamma \left|\tau \right|\right)\right)}{\left(\gamma -\alpha \right)\left(\gamma -\beta \right)\left(\beta \left(\gamma +\alpha \right)-\alpha \left(\gamma +\beta \right)\right)} \end{array}$$

The cross-covariance functions between the input and output are determined by the following expressions (17)

(35) $$\begin{array}{l} R_{xy}\left(\tau \right)=\frac{\sqrt{\gamma \left(\gamma +\alpha \right)\left(\beta +\gamma \right)}}{\left(\gamma +\alpha \right)\left(\gamma +\beta \right)\sqrt{\beta \left(\gamma +\beta \right)+\alpha \left(\gamma -\alpha \right)}}\times \\ \times \left\{ \begin{array}{l} \beta \left(\gamma -\beta \right)\left[\exp\left(-\alpha \tau \right)-\frac{2\alpha }{\alpha +\gamma }\exp\left(-\gamma \tau \right)\;\;\right]\;-\\ -\alpha \left(\gamma -\alpha \right)\left[\exp\left(-\beta \tau \right)-\frac{2\alpha }{\left(\gamma +\beta \right)}\exp\left(-\gamma \tau \right)\right],\;\tau \geq 0\\ \beta \left(\alpha +\gamma \right)\exp\left(\alpha \tau \right)\;-\left(\gamma +\alpha \right)\alpha \exp\left(\beta \tau \right)\;\;\;\;\;\;\;\;\;\;\;\;,\;\tau <0 \end{array}\right. \end{array}$$

(36) Ryx(τ)=γ(γ+α)(β+γ)(γ+α)(γ+β)β(γ+β)+α(γ−α)××{β(α+γ)exp(−ατ) −(γ+α)αexp(−βτ) ,         τ≥0   β(γ−β)[exp(ατ)−2αα+γexp(γτ)  ] −−α(γ−α)[exp(βτ)−2α(γ+β)exp(γτ)]        ,        τ<0

**Figure 5:**
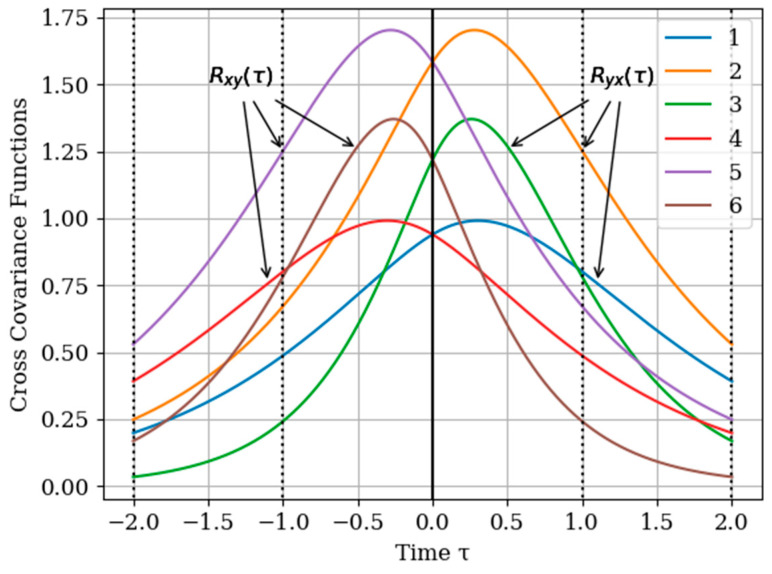
Cross-covariance functions.

Figure 5 shows the graphs of the cross covariance function $R_{xy}\left(\tau \right)$ and $R_{yx}\left(\tau \right)$ following (35) and (36) for various values of the parameters α and β. The curves are calculated for the parameters: curve 1—α=2,β=1,γ=3/8; curve 2—α=4,β=1,γ=3/8; curve 3—α=4,β=2,γ=3/4 for $R_{yx}\left(\tau \right)$ and curve 4—α=2,β=1,γ=3/8; curve 5—α=4,β=1,γ=3/8; curve 6—α=4,β=2,γ=3/4 for $R_{xy}\left(\tau \right)$.

As can be seen, the cross -covariance functions are odd, and their maxima are shifted to the points τ=−0.3 and τ=0.3 for $R_{xy}\left(\tau \right)$ and $R_{yx}\left(\tau \right)$, respectively. In general, when the value of parameters α,β,γ of cross-covariance functions $R_{xy}\left(\tau \right),R_{yx}\left(\tau \right)$ increase, their maxima are decreasing. This is due to the fact that the realizations of the input and output processes have wider spectrums Note that all the curves are smoother than those in Figure 2. This is explained by the fact that both processes x(t),y(t) are non-Markovian.

**Figure 6:**
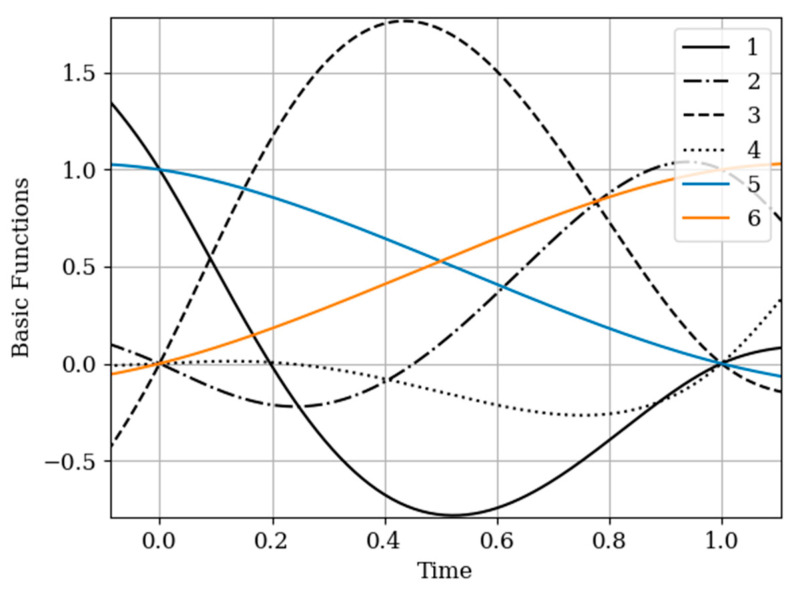
Basic functions of Section 5.2 Example 2.

**Figure 7:**
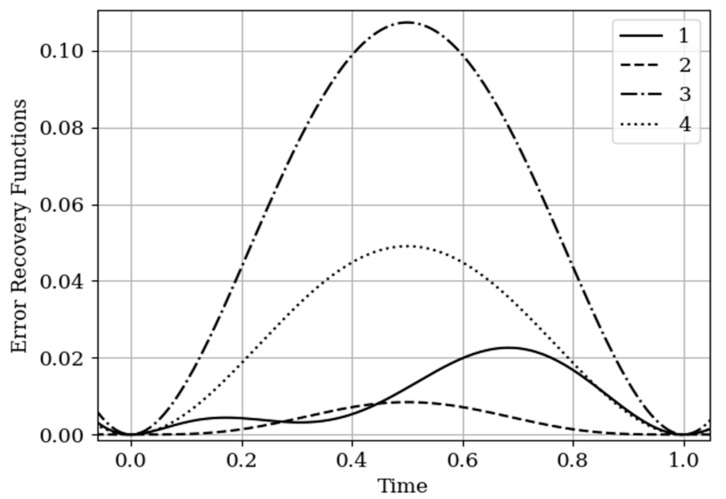
Recovery errors in Section 5.2 Example 2.

The results of calculations of basic functions and recovery errors are shown in Figure 6 and Figure 7. The values of the selected parameters are as follows: The number of samples involved in the recovery of realizations is the same: $N^{\left(x\right)}=N^{\left(y\right)}=2;$ the samples are located at the same points: T1(x)=T1(y)=0.0;T2(x)=T2(y)=1.0;α=2,β=2;γ=38;σx2=1>σy2.

In Figure 6, the basic functions of the multidimensional algorithm (curves 1–4) and one-dimensional algorithm (curves 5 and 6) are observed.

As in the previous example, covariance functions and elements of the inverse matrix influence the basic functions. The difference is explained by non-Markovian characteristics of the output process.

The results of calculations of recovery errors are shown in Figure 7. The Curves 1–4 are characterized by the same parameters as in Figure 6. When the basic functions change, the error recovery functions must also change. Comparison of the curves in Figure 4 and Figure 7 shows that the maximum error values differ significantly. This fact is explained by the greater smoothness of the studied processes in this example compared to the processes in Section 5.1 Example 1 (see more about this effect in [13,14,16]). In addition, note that the curve 1 is asymmetric compared to curve 3. This is explained because the influence of the output process determines the reconstruction of the process at the input by means of the cross-covariance function. Meanwhile, curve 2 is a symmetric function, because the cross-covariance function $R_{yx}\left(\tau \right)$ influences the reconstruction to a lesser extent.

In Section 5.1 Example 1 and Section 5.2 Example 2, the processes at the input and output of the linear system are different in the time structure: The process y(t) is more smoothed compared to the process x(t). The results of restoration errors calculations in Section 5.1 Example 1 and Section 5.2 Example 2 show that the degree of influence of additional samples of one process on the restoration quality of another process is different. Specifically, when the process is more smoothed, then its positive influence on the restoration quality of another process is significantly higher than in the other situation. (see differences between curves 1 and 3, 2 and 4 in Figure 4 and Figure 7).

The option considered in the first two examples of Section 5, in addition to theoretical, is of practical interest. We repeat that the proposed method refers to the case when the transmitted messages must have a statistical relationship. In telemetry systems, such messages are transmitted over different channels. It is quite possible that a message described by the simplest covariance function (in our model this is an input process) must be reconstructed with greater accuracy. Then, naturally, the message samples with a more complex covariance function (this is an output process) will play an auxiliary role.

### 5.3. Example 3. Displacement of the Auxiliary Sample within the Sampling Interval of the Main Component

Again, consider the system studied in Section 5.1 Example 1. That means there is a system of one RC circuit with a parameter β=1. A Markov process x(t) with a parameter α=2 acts at its input. There are three important differences: (1) The input x(t) is an auxiliary process, (2) the set of samples $X\left(T^{\left(x\right)}\right)$ consists of one sample $x\left(T_{1}^{\left(x\right)}\right)$, and (3) the location of this sample is changed within sampling interval $T_{2}^{\left(y\right)}-T_{1}^{\left(y\right)}$ of the main restored component y(t). The output process still has two samples $y\left(T_{1}^{\left(y\right)}\right)=0.0,y\left(T_{2}^{\left(y\right)}\right)=1.0$. All characteristics of this example are described by the formulas (28)–(32). For this simple variant, we specify the relations (14)–(16):

(37) $$K_{12}\left(t,T^{\left(x\right)}.T^{\left(y\right)}\right)=\left[\begin{array}{ccc} \langle \dot{x}\left(t\right)\dot{x}\left(T_{1}^{\left(x\right)}\right)\rangle & \langle \dot{x}\left(t\right)\dot{y}\left(T_{1}^{\left(y\right)}\right)\rangle & \langle \dot{x}\left(t\right)\dot{y}\left(T_{2}^{\left(y\right)}\right)\rangle \\ \langle \dot{y}\left(t\right)\dot{x}\left(T_{1}^{\left(x\right)}\right)\rangle & \langle \dot{y}\left(t\right)\dot{y}\left(T_{1}^{\left(y\right)}\right)\rangle & \langle \dot{y}\left(t\right)\dot{y}\left(T_{2}^{\left(y\right)}\right)\rangle \end{array}\right]$$

(38) $$K_{21}\left(T^{\left(x\right)},T^{\left(y\right)},t\right)=\left[\begin{array}{cc} \langle \dot{x}\left(T_{1}^{\left(x\right)}\right)\dot{x}\left(t\right)\rangle & \langle \dot{x}\left(T_{1}^{\left(x\right)}\right)\dot{y}\left(t\right)\rangle \\ \langle \dot{y}\left(T_{1}^{\left(y\right)}\right)\dot{x}\left(t\right)\rangle & \langle \dot{y}\left(T_{1}^{\left(y\right)}\right)\dot{y}\left(t\right)\rangle \\ \langle \dot{y}\left(T_{2}^{\left(y\right)}\right)\dot{x}\left(t\right)\rangle & \langle \dot{y}\left(T_{2}^{\left(y\right)}\right)\dot{y}\left(t\right)\rangle \end{array}\right]$$

(39) $$K_{22}\left(T^{\left(y\right)},T^{\left(x\right)}\right)=\left[\begin{array}{ccc} \langle {\dot{y}}_{1}\left(T_{1}^{\left(y\right)}\right){\dot{x}}_{1}\left(T_{1}^{\left(x\right)}\right)\rangle & \langle {\dot{y}}_{1}\left(T_{1}^{\left(y\right)}\right){\dot{y}}_{1}\left(T_{1}^{\left(y\right)}\right)\rangle & \langle {\dot{y}}_{1}\left(T_{1}^{\left(y\right)}\right){\dot{y}}_{2}\left(T_{2}^{\left(y\right)}\right)\rangle \\ \langle {\dot{y}}_{2}\left(T_{2}^{\left(y\right)}\right){\dot{x}}_{1}\left(T_{1}^{\left(x\right)}\right)\rangle & \langle {\dot{y}}_{2}\left(T_{2}^{\left(y\right)}\right){\dot{y}}_{1}\left(T_{1}^{\left(y\right)}\right)\rangle & \langle {\dot{y}}_{2}\left(T_{2}^{\left(y\right)}\right){\dot{y}}_{2}\left(T_{2}^{\left(y\right)}\right)\rangle \end{array}\right]$$

Elements of matrices (37) and (38) show that cross-covariance functions have an important role in calculating reconstruction error.

**Figure 8:**
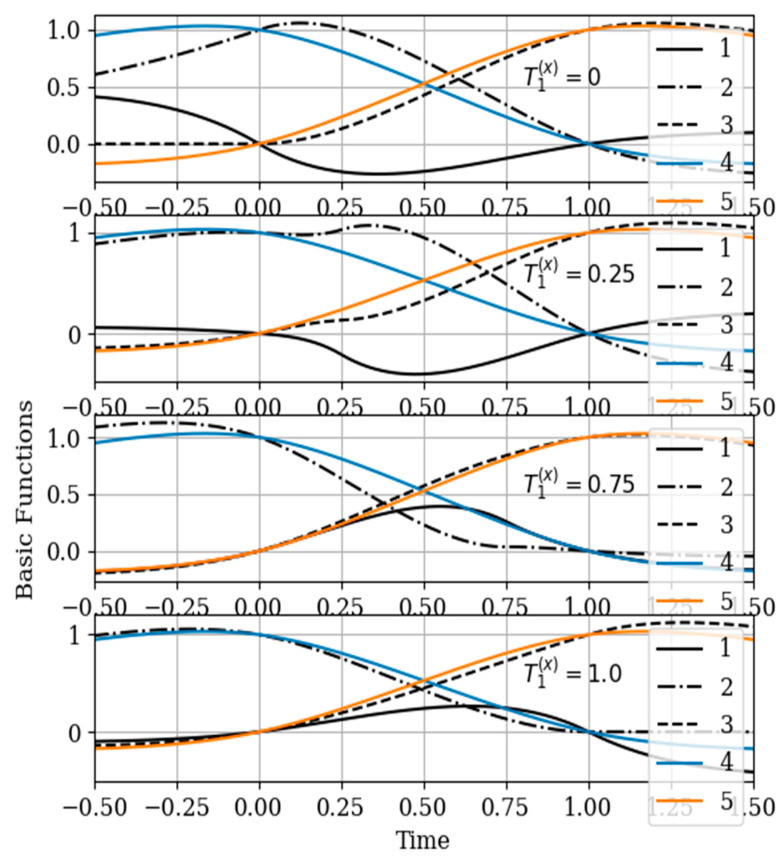
The basic functions in Section 5.3 Example 3.

**Figure 9:**
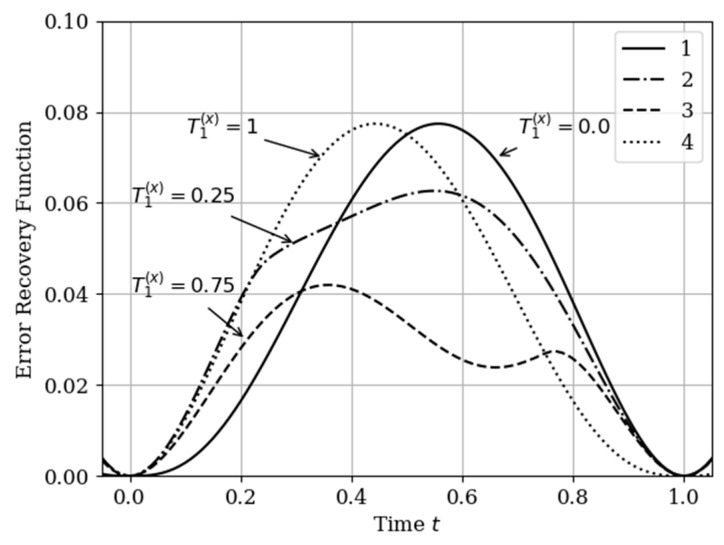
Recovery errors in Section 5.3 Example 3.

In this example, the auxiliary sample $x_{1}\left(T_{1}^{\left(x\right)}\right)$ is located at five time points: $(1)\;T_{1}^{\left(x\right)}=0;\;(2)\;T_{1}^{\left(x\right)}=0.25;\;$ $(3)\;T_{1}^{\left(x\right)}=0.75;\;(4)\;T_{1}^{\left(x\right)}=1.0$. These different points affect the shape of the basic functions as well as the course of the reconstruction error curves. The results of calculating these dependences are shown in Figure 8 and Figure 9.

It should be noted again that a realization to be restored belongs to the output process, which is characterized by the cross-covariance function in Figure 1, curve 4 in contrast to Section 5.1 Example 1 and Section 5.2 Example 2.

In Figure 8, the basic functions of the multidimensional algorithm $b_{1}^{\left(y\right)}\left(t\right)-b_{3}^{\left(y\right)}\left(t\right)$ are designated by the numbers 1, 2, 3, meanwhile the basic functions of the one-dimensional algorithm $b_{1}\left(t\right),b_{2}\left(t\right)$ are denoted by the numbers 4 and 5.

The influence of the auxiliary sampling moments $T_{1}^{\left(x\right)}$ on the reconstruction depends on the location of the maximum of the cross- covariance function $K_{xy}\left(\tau \right)$ in the interpolation region. It should be noted that the maximum of the $K_{xy}\left(\tau \right)$ covariance function (Figure 2 curve 4) is located at t=−0.45; that is, the maximum of the cross covariance function is located at $t<T_{1}^{\left(y\right)}=0$. As a consequence, $K_{xy}\left(\tau \right)$ this, the lobe of the basic function of the auxiliary sampling instant $b_{1}^{\left(y\right)}\left(t\right)$, is negative. This corresponds to the sampling moments $T_{1}^{\left(x\right)}=0;0.25$. On the other hand, the maximum of the cross-covariance function $K_{xy}\left(\tau \right)$ is in the interpolation region, that is, in the interval [0,1]. This occurs when the auxiliary sample instant is located at $T_{1}^{\left(x\right)}=0.75$. The lobe of the basic function $b_{1}^{\left(y\right)}\left(t\right)$ has positive values in this region. Finally, the auxiliary sampling instant $T_{1}^{\left(x\right)}$ is located with the second own sample, that is $T_{1}^{\left(x\right)}=T_{2}^{\left(y\right)}=1$. The influence of the mutual covariance function is located the interpolation region, that is t>1. As a result, the basic functions $b_{2}^{\left(y\right)}\left(t\right),b_{3}^{\left(y\right)}\left(t\right)$ have the same shape as the basic functions of the one-dimensional algorithm $b_{1}\left(t\right),b_{2}\left(t\right)$.

In the proposed method, with a limited number of counts, each of the counts has its own basic function. This is true even for a one-dimensional algorithm. In the multidimensional version, the situation is more complicated, since the form of the basic function is influenced by both its own samples and the samples of the auxiliary realization. Moreover, the first of them affect the form of the basic function through their own covariance function, and the second through the cross-covariance function. In addition, in both cases, the elements of the inverse covariance matrix and the temporal position of the entire set of samples play a role. The variety of these factors makes it difficult to comment on the form of basic functions (see curves in Figure 8). One can only assert the following: The article contains an analytical expression that defines the form of basic functions in general; the specified types of basic functions in all cases provide a minimum of recovery errors.

In Figure 9, there are four error recovery curves when the auxiliary sample $x_{1}\left(T_{1}^{\left(x\right)}\right)$ is located at four different instants: Curve 1—$T_{1}^{\left(x\right)}=0;$ curve 2—$T_{1}^{\left(x\right)}=0.25;$ curve 3—$T_{1}^{\left(x\right)}=0.75;$ curve 4—$T_{1}^{\left(x\right)}=1.0$.

In Figure 9 shows the influence of the auxiliary sampling moments on the reconstruction quality $T_{1}^{\left(x\right)}$. When the auxiliary sample $T_{1}^{\left(x\right)}$ is located at some point in the own samples $T_{1}^{\left(y\right)},T_{2}^{\left(y\right)}$, the error recovery is quantitatively equal $\max\left({\tilde{\sigma }}^{2}\left(t\right)\right)=0.078$, as can be seen in curves 1 and 4. When the auxiliary sample is displaced $x_{1}\left(T_{1}^{\left(x\right)}\right)=1.0$ (curve 4), the effect of the cross-covariance function $K_{xy}\left(\tau \right)$ is zero, because the influence of the auxiliary sample goes to the extrapolation interval. The different locations of the maximum error (curves 1 and 4) is explained by locating the maximum of the cross-covariance function $K_{xy}\left(\tau \right)$ at the instants $T_{1}^{\left(x\right)}=0;1$ (Figure 9). For example, when the cross-covariance function $K_{xy}\left(\tau \right)$ is at $T_{1}^{\left(x\right)}=0$, curve 1 is tilted to the right (Figure 9). This is explained by the influence of the maximum of the cross-covariance function $K_{xy}\left(\tau \right)$ manifesting itself in the region close to the sampling instant $T_{1}^{\left(y\right)}=0$. On the other hand, when the sampling moments are located in the interpolation region, the error reconstruction is reduced according to the fact that the maximum of the cross-covariance function is shifted towards the sampling moment $T_{2}^{\left(x\right)}$, as observed in curve 2 and 3.

## 6. Study Cases: Reconstruction of Realizations on Several Sampling Intervals

There are three examples here with multiple sampling intervals SRA. The input process realizations are auxiliar. The realization of the output process should be restored. Each example has its own peculiarity. Section 6.1 Example 4 and Section 6.2 Example 5 are described by the same input process and system as in Section 5.1 Example 1. Section 6.1 Example 4 differs in sampling procedures: The sampling of the input realization is non-periodic; the sample of the output realization is periodic. The number of samples is equal $N^{\left(x\right)}=N^{\left(y\right)}=3$. In Section 6.2 Example 5, the sampling of the realizations of both processes is periodic, but the instance points are offsets. The number of samples is equal $N^{\left(x\right)}=N^{\left(y\right)}=6$. Section 6.3 Example 6 examines the SRA when the input process is not Markov. The number of samples is equal $N^{\left(x\right)}=N^{\left(y\right)}=7$.

### 6.1. Example 4. SRA Algorithm with Non-Periodic Sampling of Auxiliary Input Process

There is a system of one RC circuit with a parameter β=1. A Markov process x(t) with a parameter α=2 acts at its input. The option of recovering the output process at several intervals, when the procedures for sampling the processes x(t) and y(t) are different, is considered. The numbers of samples are the same, i.e., $N^{\left(x\right)}=N^{\left(y\right)}=3$. Sampling intervals of the process y(t) are periodic: $T^{\left(y\right)}=\left[T_{1}^{\left(y\right)}=0,T_{2}^{\left(y\right)}=1.0,T_{3}^{\left(y\right)}=2.0\right]$. Samples of the realization of the process x(t) are non-periodic: $T^{\left(x\right)}=\left[T_{1}^{\left(x\right)}=0.7,T_{2}^{\left(x\right)}=1.8,T_{3}^{\left(x\right)}=2.5\right]$. This is a non-trivial case, which, however, is easily studied by the applied methodology.

**Figure 10:**
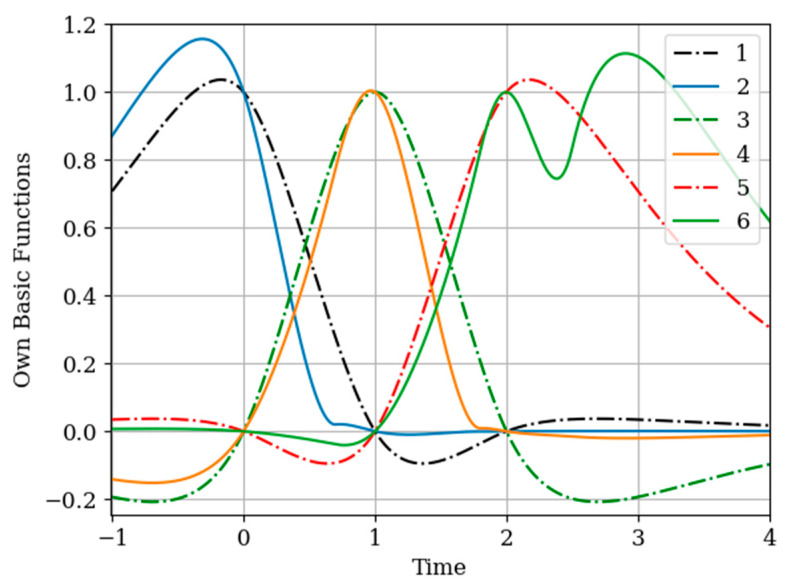
Own basic functions in Section 6.1 Example 4.

Note that the basic functions of the multidimensional algorithm $b_{4}^{\left(y\right)}\left(t\right)-b_{6}^{\left(y\right)}\left(t\right)$ (even number curves) are narrower than the basic one-dimensional functions $b_{1}\left(t\right)-b_{3}\left(t\right)$ (odd number curves). This is explained by the influence of the displaced cross covariance function $K_{xy}\left(\tau -T_{i}^{\left(x\right)}\right),\left(i=1,2,\ldots ,N^{\left(x\right)}\right)$ at the sampling instants $T_{i}^{\left(x\right)},\left(i=1,2,\ldots ,N^{\left(x\right)}\right)$ (Figure 10). This influence is weighted by the elements $a_{ij}$ of the inverse covariance matrix. This influence is most clearly seen in the basic function $b_{6}^{\left(y\right)}\left(t\right)$ (curve 6) in the extrapolation region, where there is an approximate fluctuation. This is explained by the presence of the sampling instant $T_{3}^{\left(x\right)}=2.5$.

**Figure 11:**
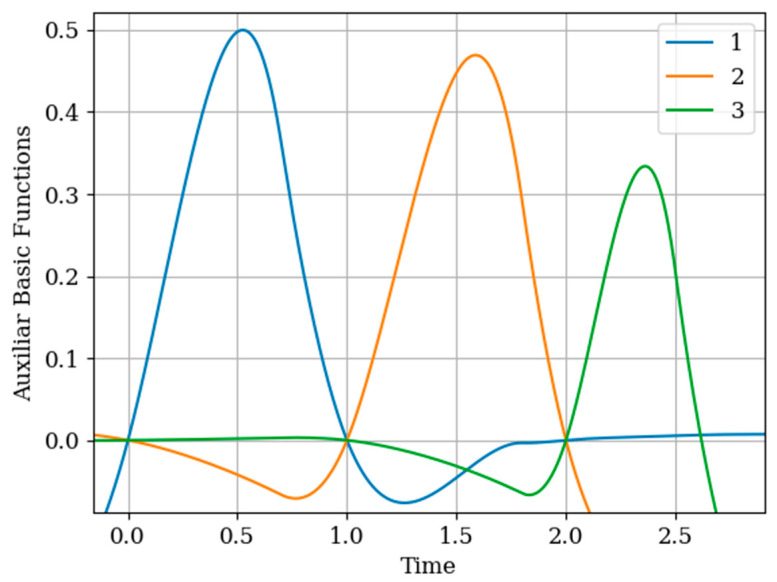
Auxiliary basic functions in Section 6.1 Example 4.

Attention should be paid to Figure 11 that shows the auxiliary main functions (curves 1–3) have a variable shape. This is explained because the sampling intervals $\Delta T^{\left(x\right)}$ between the sampling instants $T_{i}^{\left(x\right)},\left(i=1,2,\ldots ,N^{\left(x\right)}\right)$ are arbitrary. The amplitude of each basic function $b_{1}^{\left(y\right)}\left(t\right)-b_{3}^{\left(y\right)}\left(t\right)$ decreases with increasing sampling interval. This means that, as the sampling interval $\Delta T^{\left(x\right)}$ increases, the influence between the mutual covariance functions $K_{xy}\left(\tau -T_{i}^{\left(x\right)}\right),\left(i=1,2,\ldots ,N^{\left(x\right)}\right)$ decreases; this is manifested in the coefficients $a_{ij}$ in the inverse covariance matrix.

**Figure 12:**
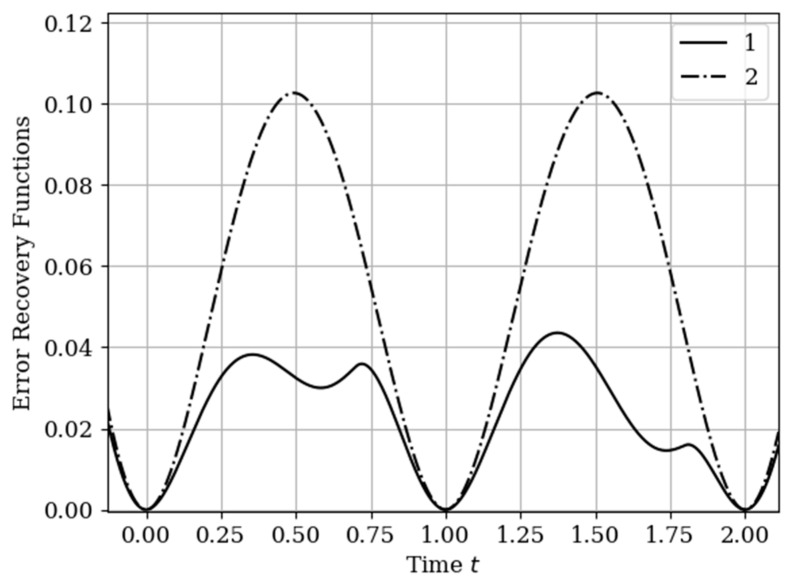
Recovery errors in Section 6.1 Example 4.

The results of calculations of recovery errors are presented in Figure 12. Curve 1 describes the recovery error using the multidimensional algorithm, and curve 2 refers to the one-dimensional version. As you can see, the character of curve 1 is different on both sampling intervals due to non-periodicity of auxiliary samples.

### 6.2. Example 5. SRA of the Realizations of Both Processes Is Periodic, but the Instance Points of the Input Are Offsets

In this example, the question of using the proposed algorithm when restoring the realization of the output process at sampling intervals 5 and 6 is considered.

The description of the system and the input process coincides with the data of Section 5.1 Example 1. All covariance functions are characterized by expressions (28), (30)–(32). The example is considered when the numbers of samples are equal $N^{\left(x\right)}=N^{\left(y\right)}=6$, and the sampling of the input x(t) and output y(t) processes occurs with different periods. So, the set of input and output processes is described by such data:

$$T^{\left(x\right)}=\left[T_{1}^{\left(x\right)}=0.75,T_{2}^{\left(x\right)}=1.75,T_{3}^{\left(x\right)}=2.75,T_{4}^{\left(x\right)}=3.75,T_{5}^{\left(x\right)}=4.75,T_{6}^{\left(x\right)}=5.75\right],$$

$$T^{\left(y\right)}=\left[T_{1}^{\left(y\right)}=0.0,T_{2}^{\left(y\right)}=1.0,T_{3}^{\left(y\right)}=2.0,T_{4}^{\left(y\right)}=3.0,T_{5}^{\left(y\right)}=4.0,T_{6}^{\left(y\right)}=5.0\right].$$

Sample sets $X\left(T^{\left(x\right)}\right),Y\left(T^{\left(y\right)}\right)$ are used to reconstruct the realization of the output process y(t).

**Figure 13:**
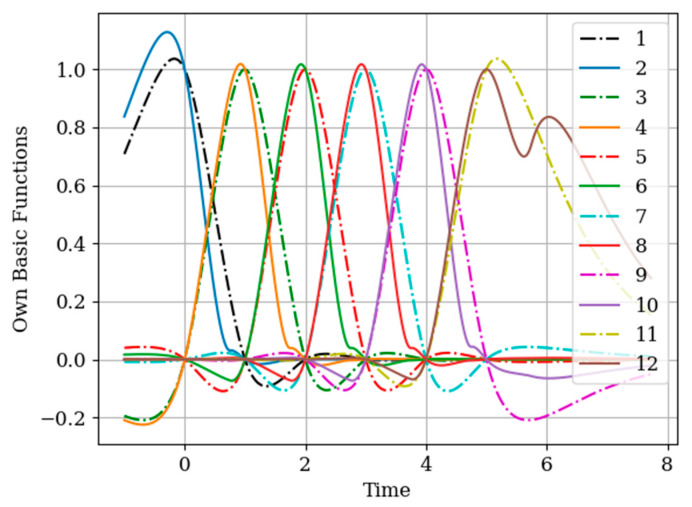
Basic functions in Section 6.2 Example 5.

The basic functions of the multidimensional algorithm $b_{7}^{\left(y\right)}\left(t\right)-b_{12}^{\left(y\right)}\left(t\right)$ (odd curves) and basic functions of the one-dimensional algorithm $b_{1}\left(t\right)-b_{6}\left(t\right)$ (even curves) are shown in Figure 13. Note that the shape of the basic functions of the multidimensional algorithm differs from the functions of the one-dimensional algorithm close to the moments of the auxiliary samples $T_{i}^{\left(x\right)},\left(i=1,2,\ldots ,N^{\left(x\right)}\right)$. This means that this difference is caused by the functions of cross-covariance $K_{xy}\left(\tau -T_{i}^{\left(x\right)}\right),\left(i=1,2,\ldots ,N^{\left(x\right)}\right)$ in the instants $T_{i}^{\left(x\right)},\left(i=1,2,\ldots ,N^{\left(x\right)}\right)$ (as one can see in Figure 2).

**Figure 14:**
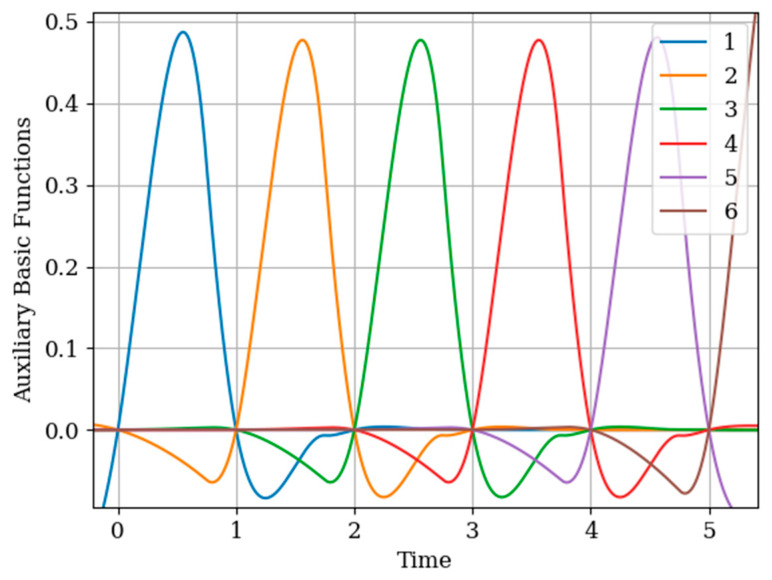
Auxiliary basic functions in Section 6.2 Example 5.

As can be seen in Figure 14, the form in the interpolation region of the auxiliary basic function $b_{1}^{\left(y\right)}\left(t\right)-b_{6}^{\left(y\right)}\left(t\right)$ is determined primarily from the cross-covariance function $K_{xy}\left(\tau -T_{i}^{\left(x\right)}\right),\left(i=1,2,\ldots ,N^{\left(x\right)}\right)$ that determines the $K_{xy}\left(\tau -T_{i}^{\left(x\right)}\right),\left(i=1,2,\ldots ,N^{\left(x\right)}\right)$, as appropriate. Note that the auxiliary basic functions $b_{1}^{\left(y\right)}\left(t\right)-b_{6}^{\left(y\right)}\left(t\right)$ have a smaller amplitude than the own $b_{7}^{\left(y\right)}\left(t\right)-b_{12}^{\left(y\right)}\left(t\right)$ and one-dimensional $b_{1}\left(t\right)-b_{6}\left(t\right)$ basic functions. This is explained by the elements of the inverse matrix $a_{ij}$. The auxiliary basic function $b_{6}^{\left(y\right)}\left(t\right)$ has a different form than the basic functions $b_{1}^{\left(y\right)}\left(t\right)-b_{5}^{\left(y\right)}\left(t\right)$. The reason for this is that all coefficients are positive for the auxiliary sample $T_{6}^{\left(x\right)}=5.75$. That means covariance functions and cross-covariance functions are summed.

**Figure 15:**
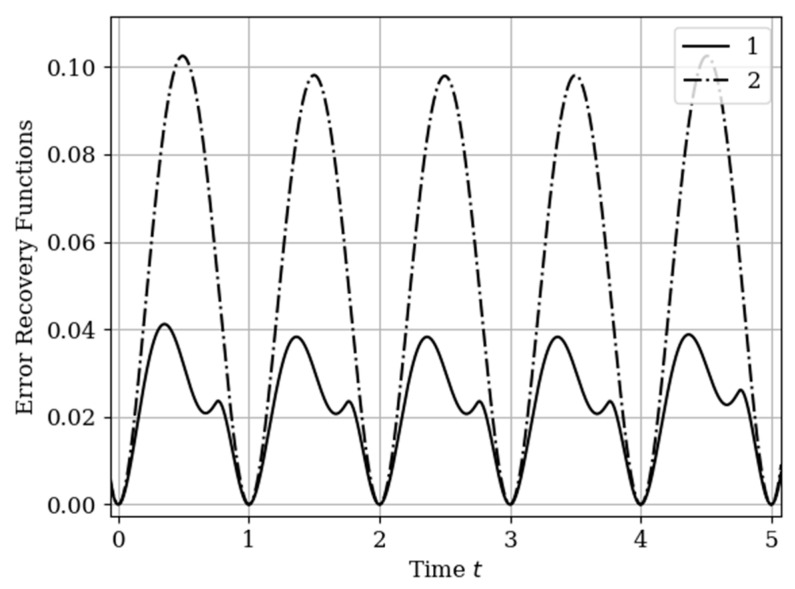
Recovery errors in Section 6.2 Example 5.

The recovery error of the output process y(t) by multidimensional and one-dimensional algorithms is illustrated in Figure 15. Curve 1 characterizes the recovery using a multidimensional algorithm. Curve 2 relates to a one-dimensional algorithm. The influence of the displacement of the auxiliary samples with respect to the own samples $T_{i}^{\left(y\right)},\left(i=1,2,\ldots ,N^{\left(y\right)}\right)$ is observed. This means that the maximums of the cross-covariance functions are located the interpolation region. This location corresponds to the minimum of the reconstruction error function, that is $t=\;0.75+T_{i}^{\left(y\right)},\left(i=1,2,\ldots ,N^{\left(y\right)}\right)$. There is a small smoothed minimum at the highs of curve 2 in the middle of the total interval. This effect for non-Markov processes is described in the analysis of a one-dimensional algorithm [13,14,16]. In this example, the difference in the maxima of the one-dimensional curves is insignificant. On curve 1, this effect is seen by the dependence among their samples.

### 6.3. Example 6. SRA When the Input Process Is Non–Markovian

Consider another example, which is an analogue of Section 5.2 Example 2. Here, the system is an RC circuit with a parameter γ, and the input process is formed from white noise by two consecutive RC circuits with parameters α,β. Covariance functions are defined by expressions (34)–(36). The input process x(t) here is non-Markovian. Input and output processes are sampled as follows:

$$T^{\left(x\right)}=\left[T_{1}^{\left(x\right)}=0.6,T_{2}^{\left(x\right)}=1.6,T_{3}^{\left(x\right)}=2.6,T_{4}^{\left(x\right)}=3.6,T_{5}^{\left(x\right)}=4.6,T_{6}^{\left(x\right)}=5.6,T_{7}^{\left(x\right)}=6.6\right]$$

$$T^{\left(y\right)}=\left[T_{1}^{\left(y\right)}=0.0,T_{2}^{\left(y\right)}=1.0,T_{3}^{\left(y\right)}=2.0,T_{4}^{\left(y\right)}=3.0,T_{5}^{\left(y\right)}=4.0,T_{6}^{\left(y\right)}=5.0,T_{7}^{\left(y\right)}=6.0\right]$$

As can be seen, the number of samples is different and equal to 7. Input samples are delayed for a while t=0.6. A realization of the output process y(t) is reconstructed.

**Figure 16:**
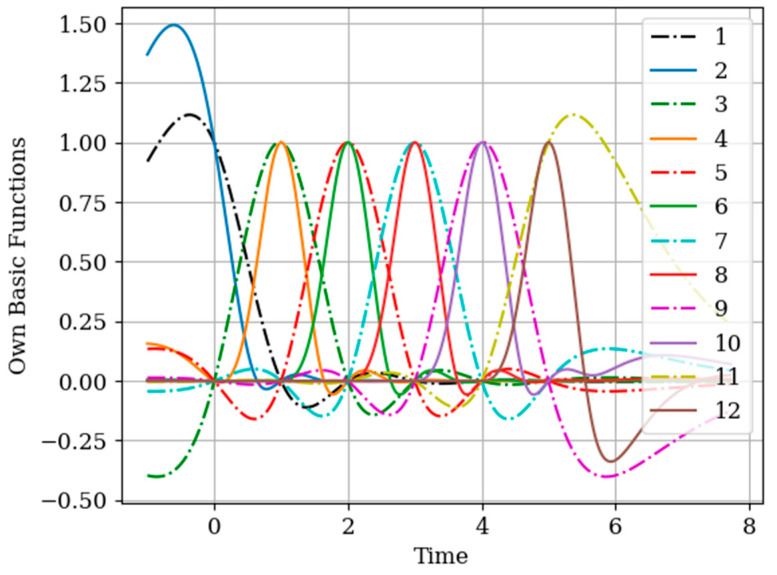
Own basic functions in Section 6.3 Example 6.

In Figure 16, the basic functions of the multidimensional $b_{7}^{\left(y\right)}\left(t\right)-b_{12}^{\left(y\right)}\left(t\right)$ (odd curves) and one-dimensional algorithm $b_{1}\left(t\right)-b_{6}\left(t\right)$ (even curves) are compared. Note that the maximum of the basic functions of the multidimensional and one-dimensional algorithm corresponds to the sampling instant $T_{i}^{\left(y\right)},\left(i=1,2,\ldots ,N^{\left(y\right)}\right)$. This means that there is a greater of the covariance functions $K_{y}\left(\tau -T_{i}^{\left(y\right)}\right),\left(i=1,2,\ldots ,N^{\left(y\right)}\right)$, which are weighted by the elements of the inverse covariance matrix $a_{ij}$, as observed in Formula (27). Another feature to note is that the basic functions of the multidimensional algorithm $b_{7}^{\left(y\right)}\left(t\right)-b_{12}^{\left(y\right)}\left(t\right)$ are narrower than the functions of the one-dimensional algorithm $b_{1}\left(t\right)-b_{6}\left(t\right)$. This is because all the cross-covariance functions $K_{xy}\left(\tau -T_{i}^{\left(x\right)}\right),\left(i=1,2,\ldots ,N^{\left(x\right)}\right)$ influence the recovery of the samples $T_{i}^{\left(y\right)},\left(i=1,2,\ldots ,N^{\left(y\right)}\right)$.

**Figure 17:**
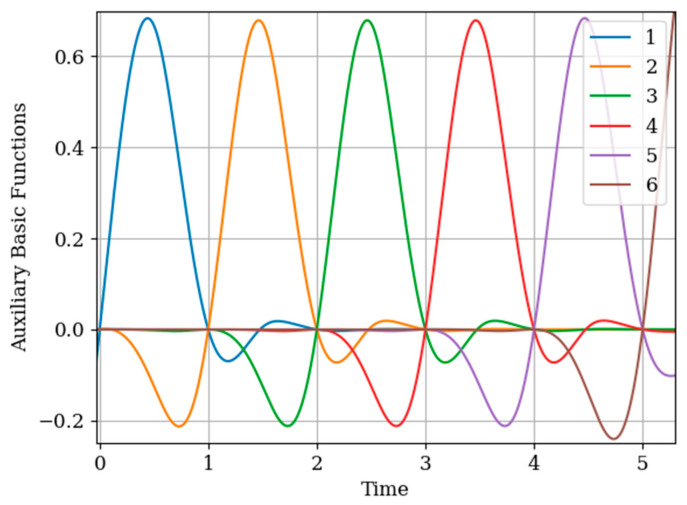
Basic functions in Section 6.3 Example 6.

In Figure 17, the auxiliary basic functions $b_{1}^{\left(y\right)}\left(t\right)-b_{6}^{\left(y\right)}\left(t\right)$ of the multidimensional algorithm are observed. Comparing the results with Figure 15, the amplitude of the functions $b_{1}^{\left(y\right)}\left(t\right)-b_{6}^{\left(y\right)}\left(t\right)$ in Figure 17 is greater. This is related to the cross-covariance function $K_{xy}\left(\tau -T_{i}^{\left(x\right)}\right),\left(i=1,2,\ldots ,N^{\left(x\right)}\right)$, which is manifested in the elements of the inverse covariance matrix $a_{ij}$. To explain the last basic function $b_{6}^{\left(y\right)}\left(t\right)$ of the last instant of the auxiliary sample $T_{6}^{\left(x\right)}=5.6$ concentrates the influence in an additive form (that is, the coefficients of the inverse matrix $a_{ij}$ are positive in this last auxiliary sampling instant) of the covariance function $K_{y}\left(\tau \right)$ and the mutual covariance function $K_{xy}\left(\tau \right)$.

**Figure 18:**
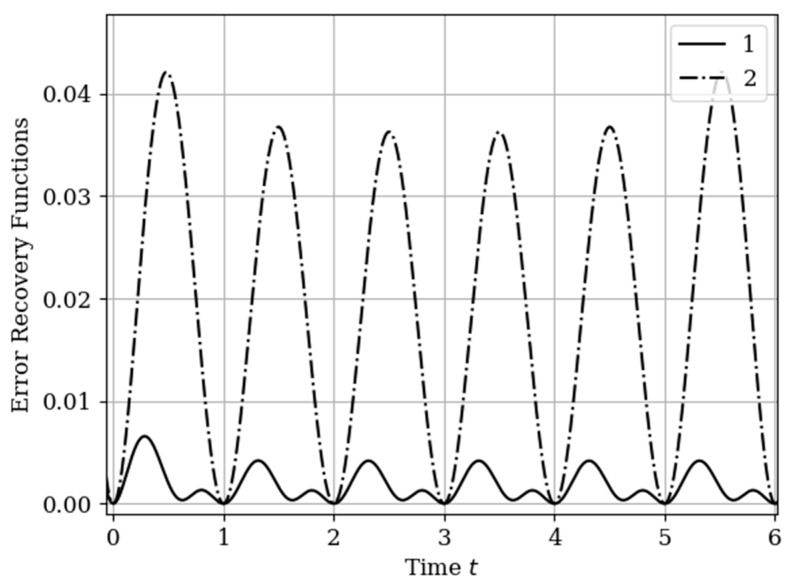
Recovery errors in Section 6.3 Example 6.

The form in Figure 18 of the curves shows an analogy with Figure 15 in Section 6.2 Example 5. The main differences in (18) (with a comparison of Figure 15) are associated with a significant decrease in the values of the errors n and the asymmetric nature of the curves related to multivariate recovery. The reasons are obvious: (1) The output process y(t) is smoother and (2) the shift of the samples of the set $T^{\left(x\right)}$ as compared with the samples of the set $T^{\left(y\right)}$, and with the size of the sampling interval, is insignificant (0.15). Curve 1 shows the effect of reducing the error in the center between the extreme samples. Obviously, this is a reflection the greater statistical relationship between samples in the considered non-Markov process.

The increase in the quality of restoration (in Figure 18) is physically explained by the fact that in the known method when restoring realizations, only its own samples are used. In the proposed method, the number of samples participating in the reconstruction is increased due to samples from another, statistically related realization. Moreover, the number of additional samples can be arbitrary. It is obvious that the restoration of realizations from a larger number of samples leads to an increase in the quality of restoration.

## 7. Conclusions

The problem investigated in the article work to the problem of sampling—recovery of two-dimensional Gaussian processes. The dimensionality of the problem is not limited by the presence of two random processes at the input and output of the linear system, since, in addition to them, the problem includes two sets of samples fixed in the realizations of these processes. The algorithm developed differs in that the reconstruction of the realizations of both components, or one of them, is carried out on the basis of two sets of samples. This means that the recovery occurs not only with the participation of its own realization samples, but with the realization samples of another component. The considered examples illustrate some applications of the proposed algorithm. They studied the options when the following changes: (1) The type and input of the system, (2) the number of intervals on which the restoration is performed, (3) as well as the number of auxiliary samples involved in the functioning of the multidimensional algorithm.

In all cases, there are basic functions and error recovery functions. These functions are optimal and characterize the estimation of yields using the recovery algorithm studied. These reconstruction characteristics allow us to demonstrate the advantage of using the algorithm based on the quality of the reconstruction. This result will be used as long as the random processes have a statistical dependency.

## Appendix A. Formulas for Conditional Statistical Characteristics of Multidimensional Gaussian Random Variables

Application of the conditional average rule (CMR) to the problem under consideration requires knowledge of the conditional characteristics for the Gaussian process. In mathematical statistics, a general expression is known that allows one to determine the conditional expectation vector and the conditional covariance matrix of a k-dimensional random variable based on a priori information about a n-dimensional Gaussian variable (n>k). The following general formulas fully fall under the CMR as applied to a multidimensional Gaussian random variable.

We give these formulas using the simplest notation [23]. In the text of the article, these formulas are concretized for the studied problem.

Consider a n-dimensional Gaussian variable

(A1) $$z={\left[z_{1},z_{2}\right]}^{T},\;z_{1}={\left[z_{1},z_{2},\ldots ,z_{k}\right]}^{T},z_{2}={\left[z_{k+1},z_{k+2},\ldots ,z_{n}\right]}^{T}$$

which is completely described by the vector of mathematical expectations

(A2) $$\langle z\rangle ={\left[\langle z_{1}\rangle ,\langle z_{2}\rangle \right]}^{T}$$

and covariance matrix K of the column vector $z={\left[z_{1},z_{2}\right]}^{T}$:

(A3) $$K=\left[\begin{array}{cc} K_{11} & K_{12}\\ K_{21} & K_{22} \end{array}\right]$$

where $K_{11},K_{22}$ are the covariance matrices of vectors $z_{1}$ and $z_{2}$, respectively; $K_{12},K_{21}$—matrices of cross covariance between vectors $z_{1}$ and $z_{2}$.

Suppose that the vector $z_{2}$ is fixed, then the vector $z_{1}$ becomes conditional with respect to the vector $z_{2}$. Its statistical characteristics are denoted by tildes. Then, the vector of conditional mathematical expectations $\langle {\tilde{z}}_{1}\rangle$ and the matrix of conditional covariance $\tilde{K}$ are written in the form [22,23]:

(A4) $$\langle {\tilde{z}}_{1}\rangle =\langle z_{1}\rangle +K_{12}K_{22}^{-1}\left(z_{2}-\langle z_{2}\rangle \right)$$

(A5) $$\tilde{K}=K_{11}-K_{12}K_{22}^{-1}K_{21}$$
